# Translational control of Bcl-2 promotes apoptosis of gastric carcinoma cells

**DOI:** 10.1186/s12885-020-07711-6

**Published:** 2021-01-05

**Authors:** Shuangfen Tao, Jianchun Gu, Qing Wang, Leizhen Zheng

**Affiliations:** Oncology Department, Xin Hua Hospital affiliated To Shanghai Jiao Tong University School of Medicine, 1665 Kongjiang Road, Shanghai, 200092 China

**Keywords:** Bcl-2, Gastric carcinoma (GC), miR-383, Chemotherapy

## Abstract

**Background:**

Anti-apoptotic protein Bcl-2 plays a substantial role in the carcinogenesis, whereas the regulation for Bcl-2 in gastric carcinoma (GC) is poorly understood. Specifically, a role of microRNA (miR)-383 in the control of Bcl-2 has not been shown in GC and thus addressed in the current study.

**Methods:**

We investigated the levels of miR-383 and Bcl-2 in 50 GC specimens, and compared them with patients’ clinical characteristics. Bioinformatics analyses and luciferase-reporter assay were applied for analyzing the relationship between Bcl-2 and miR-383. An CCK assay was used to determine the survival of Fluorouracil-treated GC cells, and apoptosis of GC cells was assessed by flow cytometric FITC Annexin V apoptosis detection assay and expression of apoptosis-associated proteins.

**Results:**

The levels of miR-383 were lower while the levels of Bcl-2 levels were higher in GC specimens, compared to tissue from the adjacent non-tumor region. Low miR-383 and high Bcl-2 seemed to be associated with high malignancy and metastasis. In GC specimens, the levels of Bcl-2 and miR-383 inversely correlated. The overall survival of miR-383-low cases was poorer. Mechanistically, miR-383 targeted the 3′-UTR of Bcl-2 mRNA to inhibit its protein translation. Overexpression of miR-383 downregulated Bcl-2, resulting in reduced survival of Fluorouracil-treated GC cells. Similar conclusion was drawn through analysis of published database.

**Conclusion:**

MiR-383 reduces survival of Fluorouracil-treated GC cells through downregulating of Bcl-2.

**Supplementary Information:**

The online version contains supplementary material available at 10.1186/s12885-020-07711-6.

## Background

Cancer-related death for gastric carcinoma (GC) is especially high in China [1]. Chemotherapy with Fluorouracil (5-FU) is an effective adjuvant treatment for surgical resection of primary GC, which potentially improves GC patients’ survival [2]. Of note, some GCs appear to be quite resistant to 5-FU treatment, without a completely defined molecular mechanism. It is however expected to be resulting from augmentation of anti-apoptotic potential of GC cells during 5-FU treatment [3–5], likely through altered expression of certain apoptosis-associated proteins, such as Bcl-2 [6].

In researches to determine the molecular carcinogenesis of GC [7]. Strong evidence supports contribution of abnormal microRNAs (miRNAs) in the carcinogenesis of GC [8–10]. MiRNA is a group of small non-coding RNA of comprising of 18–23 nucleotides, which typically regulate target genes through competitive binding on the 3′-untranslated region (3′-UTR) of their mRNA [11, 12], leading to altered protein translation and degradation. Therefore, miRNAs are crucial for many biological events including tumorigenesis [8–10, 13]. Interestingly, miR-383 has been shown to inhibit retinal pigment epithelial cell viability and to promote apoptosis and ROS formation likely through B-cell lymphoma 2 (Bcl-2) and peroxiredoxin 3 [14]. In another study, miR-383 upregulation was shown to prevent propofol-induced apoptosis of hippocampal neuron and cognitive impairment via Bcl-2 [15]. Very recently, miR-383 has been shown downregulated in GC and to be associated with poor prognosis [16, 17]. Nevertheless, the underlying mechanisms are not fully understood and addressed in the current study.

## Methods

### Specimen of patient tissues

In this research, we used clinically and histologically diagnosed 50 resected specimens of GC (paired normal gastric tissue (NT) with GC; TNM stage varied from II to IV) at the Xin Hua Hospital from 2011 to 2014. All the patients had received tumor resection and following chemotherapy with 5-FU. The relationship between the clinicopathological parameters of these GC patients and analyzed factors was summarized in Table 1. The access of the clinical materials for research has obtained prior written consent from both patients and the authorization from the Institutional Research Ethics Committee of Xin Hua Hospital.

**Table 1:** Multivariate analysis for Bcl-2 and miR-383 with clinicopathological parameters in GC patients

Parameter	Bcl-2-high	Bcl-2-low	***P*** value	miR-383-high	miR-383-low	*P* value
**Age (years)**						
< 60	12 (24%)	11 (22%)	NS	9 (18%)	14 (28%)	NS
> 60	13 (26%)	14 (28%)		16 (32%)	11 (22%)	
**Sex**						
Male	18 (36%)	15 (30%)	NS	14 (28%)	19 (38%)	NS
Female	7 (14%)	10 (20%)		11 (22%)	6 (12%)	
**Lauren’s classification**						
intestine	17 (34%)	22 (44%)	< 0.05	23 (46%)	16 (32%)	< 0.05
diffuse	8 (16%)	3 (6%)		2 (4%)	9 (18%)	
**TNM stage**						
III + IV	21 (42%)	7 (14%)	< 0.05	9 (18%)	20 (40%)	< 0.05
II	4 (8%)	18 (36%)		16 (32%)	5 (10%)	
**Lymph node metastasis**						
Present	22 (44%)	15 (30%)	< 0.05	15 (30%)	22 (44%)	< 0.05
Absent	3 (6%)	10 (20%)		10 (20%)	3 (6%)	
**Tumor location**						
Upper 1/3	4 (8%)	3 (6%)	NS	3 (6%)	4 (8%)	NS
Middle 1/3	8 (16%)	9 (18%)		10 (20%)	7 (14%)	
Lower 1/3	13 (26%)	13 (26%)		12 (24%)	14 (28%)	

### Cell lines, reagents and transfection

Two human GC cell lines, AGS and SNU-5, were purchased from American Type Culture Collection (ATCC, Rockville, MD, USA). The human cell lines used are commercially sourced and do not need ethical approval. Free of mycoplasma contamination was routinely tested. We cultured these two cell lines in RPMI1640 medium (Invitrogen, Shanghai, China) with 10% fetal bovine serum (FBS; Sigma-Aldrich, Shanghai, China) in 5% CO_2_ humidified chambers under 37 °C. 5-FU (Sigma-Aldrich) was diluted into 1 mmol/l and used at a terminal concentration of 5 μmol/l to cultured cells. We purchased plasmids carrying antisense (as) for miR-383 and miR-383-mimic (5′-AGAUCAGAAGGUGACUGUGGCU-3′) (RiboBio Co., Ltd., Shanghai, China), for transfection with Lipofectamine 3000 reagent (Invitrogen). The cells were also transfected with a null plasmid as a control (null). Cell viability was assessed with an CCK-8 detection kit (Sigma-Aldrich).

### Elisa

We extracted proteins from NT or GC specimens, or from sorted or cultured cells, with a lysis buffer (RIPA, Sigma-Aldrich) on ice. The ELISA for Bcl-2 and cleavage caspase 3 (Casp3) were performed using a human Bcl-2 ELISA kit (LS-F4134, LSbio Lifespan Biosciences Inc., Seattle, WA, USA) or a human cleavage Casp3 ELISA kit (KM300, R&D Biosystem, Beijing, China).

### Quantitative RT-PCR (RT-qPCR)

We extracted total RNAs from tissue specimens or from sorted or cultured cells using miRNeasy mini kit (Qiagen, Hilden, Germany). RT-qPCR was performed subsequently using reversely transcribed cDNA and a QuantiTect SYBR Green PCR Kit (Qiagen). Primers: miR-383: forward 5′-CTCCTCAGATCAGAAGGTGATTG-3′ and reverse 5′-CTCTTTCTGACCAGGCAGTG-3′; Bcl-2: forward 5′-TCGCCCTGTGGATGACTGA-3′ and reverse 5′-CAGAGACAGCCAGGAGAAATCA-3′; GAPDH: forward: 5′-GCGAGATCCCTCCAAAATCAA-3′ and reverse 5′-GTTCACACCCATGACGAACAT-3′. Data were analyzed with 2-△△Ct method. Gene values were obtained by sequential normalization against housekeeping gene GAPDH and the corresponding experimental control.

### Prediction of MiRNA target and approval

The targets of miRNAs were predicted with the algorithms TargetScan [18]. Target constructs for 3′-UTR of wildtype Bcl-2 and another with a site mutation at miR-383-binding site were purchased from Creative Biogene (Shirley, NY, USA). The co-transfection was performed on cells using 1 μg plasmids for 24 h, followed by measurement activities of Luciferase activity with a dual-luciferase reporter gene assay kit (Promega, Shanghai, China).

### The assay of apoptosis by flow cytometry

The re-suspended cultured cells or dissociated tumor cells in PBS underwent FAC analysis to determine the percentage of Annexin V+ PI- apoptotic cells after co-labeling with FITC-conjugated Annexin V and propidium iodide (PI), using a FITC Annexin V Apoptosis Detection Kit I (Becton-Dickinson Biosciences, Beijing, China). For isolation of inflammatory cells, tumor tissue was digested with 0.25% trypsin (Sigma-Aldrich) for 40 min and incubated with an APC-conjugated anti-CD45 antibody before subjected to Flow cytometry. Inflammatory cells were CD45-positive cells, while CD45-negative cells were mainly tumor cells.

### Analysis of statistics

Experimental sample size was determined by power calculations based on α = 5 and 80% power to detect indicated difference. Expected variance of each assay is based either on our prior experience or published data. Results were assessed using appropriate statistical tests, including student t-tests and one-way ANOVA method coupled with a Bonferroni correction (GraphPad Prism 7, GraphPad Software, Inc. La Jolla, CA, USA). Spearman’s Rank Correlation Coefficients were applied for calculating Bivariate correlations. Kaplan-Meier curves were employed to determine the survival of grouped patients. Mean ± standard deviation (SD) was used to present sample values and considered significant when *p* < 0.05. For bioinformatics analysis, transcriptome RNA-sequencing (RNA-seq) data of human GC cells were obtained from the GEO data portal (https://www.ncbi.nlm.nih.gov/geo/). Four specimen database (GSE107754, GPL4133, GPL570 and GPL5639) were subjected to R software Linear Models for RNA-Seq Data (Limma) package. The differential gene expression was presented with a log2 |fold change| > 4.0 and a false discovery rate (FDR) < 0.05 as the cutoff values.

## Results

### Altered miR-383 and Bcl-2 inversely correlates in GC specimens

**Fig. 1:**
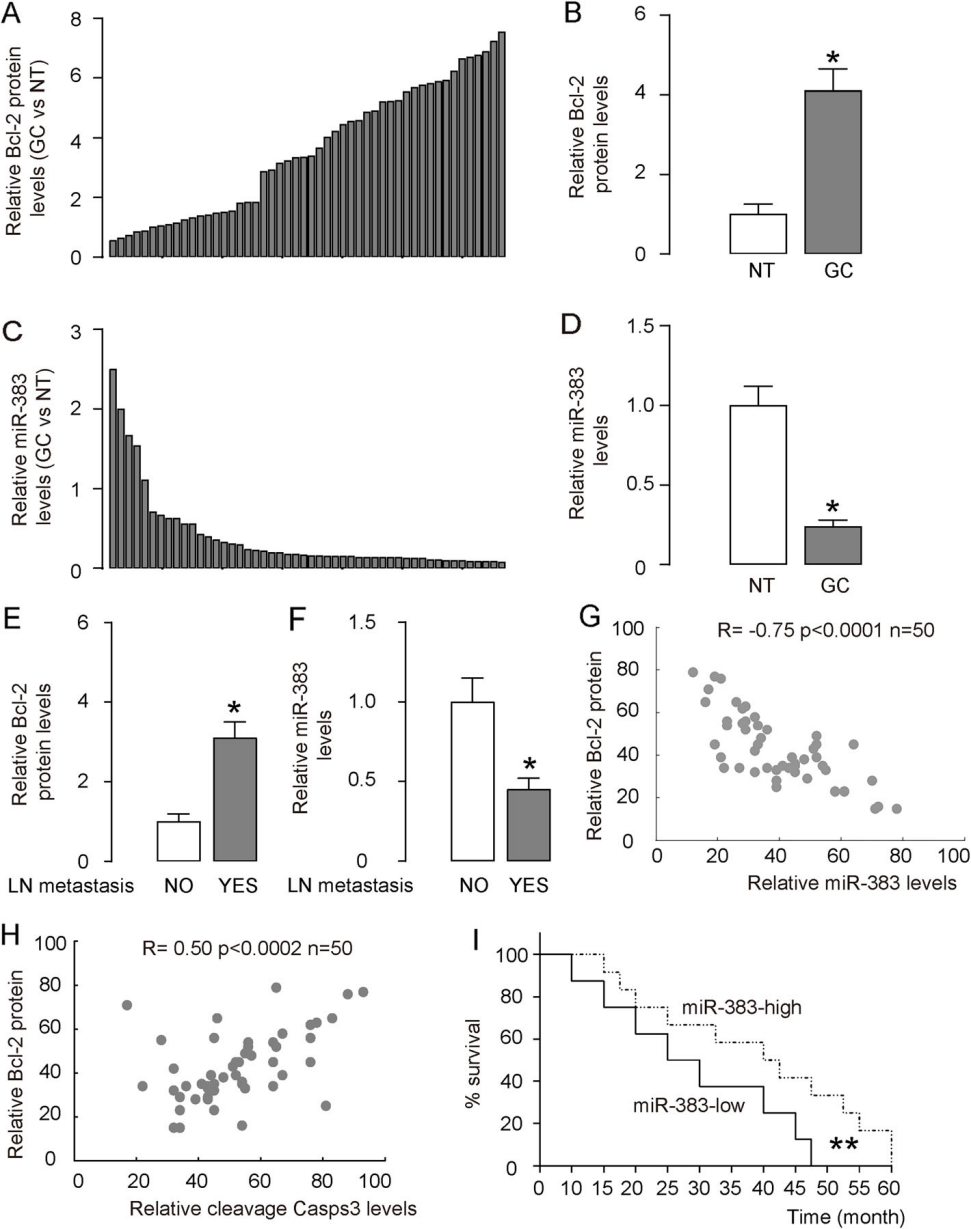
Altered miR-383 and Bcl-2 inversely correlates in GC specimens. RT-qPCR on miR-383 and ELISA for Bcl-2 were performed on paired GC and the adjacent non-tumor gastric tissue (NT) from 50 patients. **a-b** Bcl-2 levels, shown by individual values (**a**) and by mean ± SD (**b**). **c-d** miR-383 levels, shown by individual values (**c**) and by mean ± SD (**d**). For **b** and **d**, values were normalized to NT (=1). **e** ELISA for Bcl-2 in GC with or without LN metastasis. **f** RT-qPCR for miR-383 in GC with or without LN metastasis. For E and F, values were normalized to GC without LN metastasis (=1). **g** A Correlation test between relative Bcl-2 and miR-383 levels. Mean level was set up as 50. **h** A Correlation test between relative Bcl-2 and cleavage Casp3 levels. Mean level was set up as 50. **i** Kaplan-Meier curve for overall survival. *p < 0.05. **p < 0.01. *N* = 50

We analyzed specimens from 50 GC patients TNM-staged II to IV, since we wanted to analyze all cases with similar treatment (removal of GC followed with 5-FU therapy; most stage I patients were treated differently). Since we planned to analyzed Bcl-2 and miR-383 levels in GC versus paired non-tumor tissue (NT), we first excluded the effects of intra-tumoral inflammatory cells on analysis of total tumor tissue. The cancer specimens were dissociated and then underwent a FAC-sorting for a pan-leukocyte-marker CD45 by flow cytometry to compare expression levels of Bcl-2 and miR-383 in inflammatory cells (including macrophages and lymphocytes) and in tumor cells, showing that the major source of Bcl-2 and miR-383 in GC tissue is tumor cells rather than inflammatory cells (Supplementary Figure). We found that Bcl-2 protein levels in GC specimens were significantly higher (Fig. 1a-b), while miR-383 levels in GC specimens were significantly lower (Fig. 1c-d), comparing with NT. No significant difference in age, sex, tumor location was detected between Bcl-2-high and Bcl-2-low GC cases, or between miR-383-high and miR-383-low GC cases (Table 1). However, high Bcl-2 and low miR-383 appeared associated with higher TNM stages, more lymph node (LN) metastasis and differed Lauren’s classification (Table 1). Correspondingly, LN-metastatic GC carried higher Bcl-2 and lower miR-383 compared to GC without LN metastasis (Fig. 1e-f). To evaluate the relationship between Bcl-2 and miR-383, we assessed miR-383 levels and Bcl-2 protein in these 50 GC samples and detected a strong inverse correlation between the two factors (Fig. 1g). Furthermore, cleavage caspase 3 (Casp3) was also quantified in these samples and exhibited positive correlation with Bcl-2 (Fig. 1h), suggesting that Bcl-2 levels may be associated with GC apoptosis. Next, we examined if the miR-383 levels might correlate with the overall GC patients’ survival for 5 years. The miR-383-high cases (*n* = 25) and the miR-383-low cases (n = 25) were separated by the median value of all 50 cases. The overall survival of miR-383-low GC patients was obviously poorer than miR-383-high GC patients (Fig. 1i).

### MiR-383 suppresses translation of Bcl-2 via targeting 3′-UTR mRNA

**Fig. 2:**
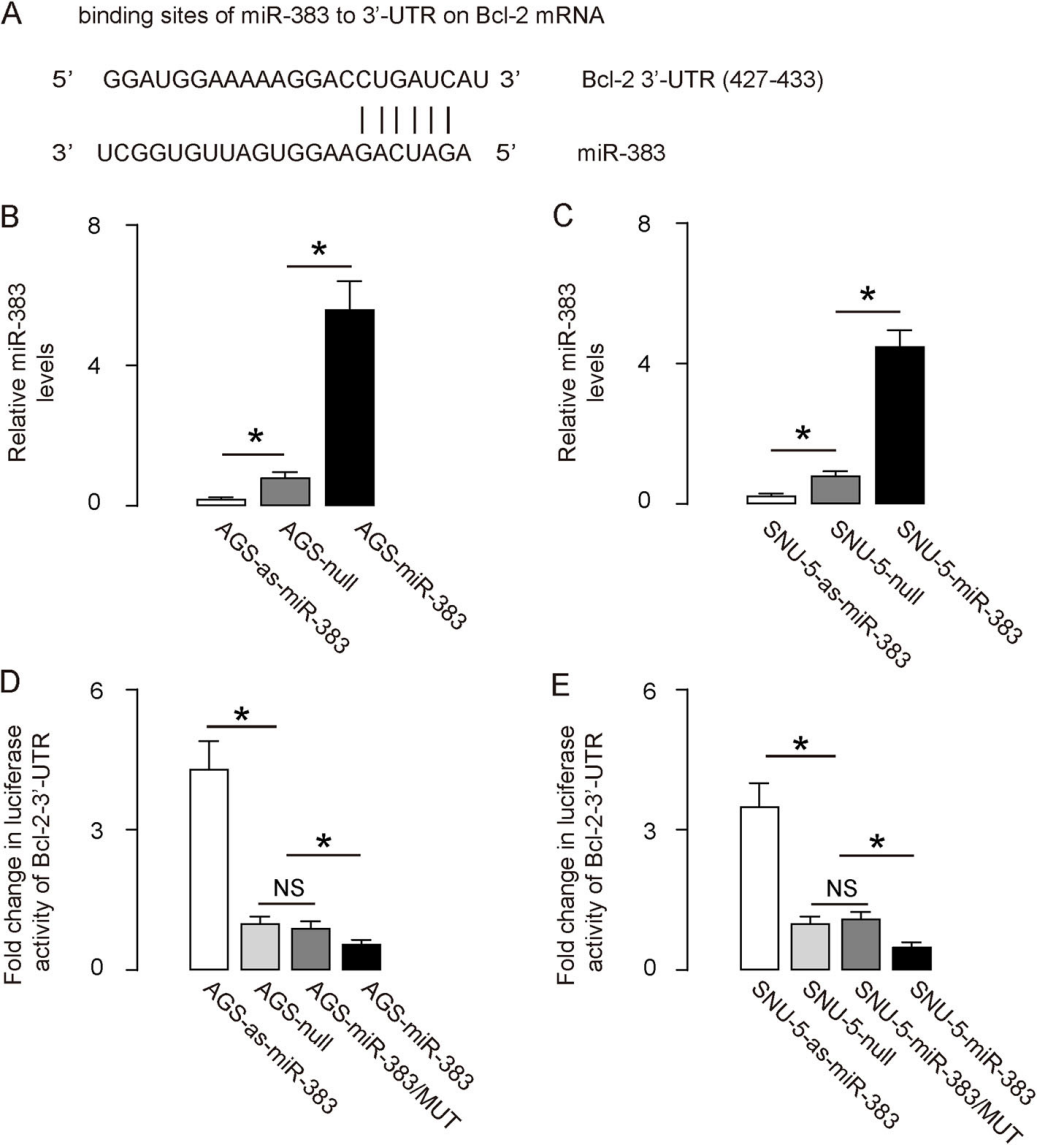
MiR-383 inhibits translation of Bcl-2 mRNA via 3′-UTR binding. **a** Bioinformatic prediction. **b-c** Overexpression or depletion of miR-383 in 2 human GC cell lines, AGS (**b**) and SNU-5 (**c**), by transfection with a miR-383-mimic, or with a plasmid carrying miR-383 antisense. Transfection with a null plasmid was used as a control. RT-qPCR was performed to assess miR-383 levels. For B and C, values were normalized to null-transfected cells (=1). (D-E) MiR-383-modified AGS (**d**) or SNU-5 (**e**) cells were transfected with wildtype or mutant Bcl-2-3’UTR for a luciferase reporter assay. For D and E, values were normalized to null only -transfected cells (=1). *p < 0.05. *N* = 5 (each experimental group contains 5 repeats)

Since our analysis on clinical samples suggest a relationship between Bcl-2 and miR-383 in GC specimens, we were eager to know whether Bcl-2 may be a direct target for miR-383. A miR-383-binding site ranged from 427th to 433th base at the 3′-UTR of Bcl-2 mRNA was predicted (Fig. 2a). To prove it, first we either overexpressed miR-383 in 2 human GC cell lines AGS and SNU-5, by transfection with plasmids carrying a miR-383-mimic (AGS-miR-383; SNU-5-miR-383), or inhibited miR-383 in these 2 lines with a miR-383 antisense (AGS-as-miR-383; SNU-5-as-miR-383). Also, the AGS and SNU-5 cells were also transfected with a plasmid carrying null sequence as a control (AGS-null; SNU-5-null). RT-qPCR was then performed to confirm the effectiveness of these miR-383-modified plasmids (Fig. 2b-c). Subsequently, we transfected miR-383-modified cells with Bcl-2 3′-UTR luciferase-reporter plasmid, with or without a site mutation on the miR-383 binding sites. The activities of luciferase were quantified in these cells, which suggested that the binding of miR-383 to 3′-UTR of Bcl-2 mRNA inhibited the translation of Bcl-2 protein (Fig. 2d-e).

### MiR-383 decreases Bcl-2 protein in cells of GC without affecting mRNA

**Fig. 3:**
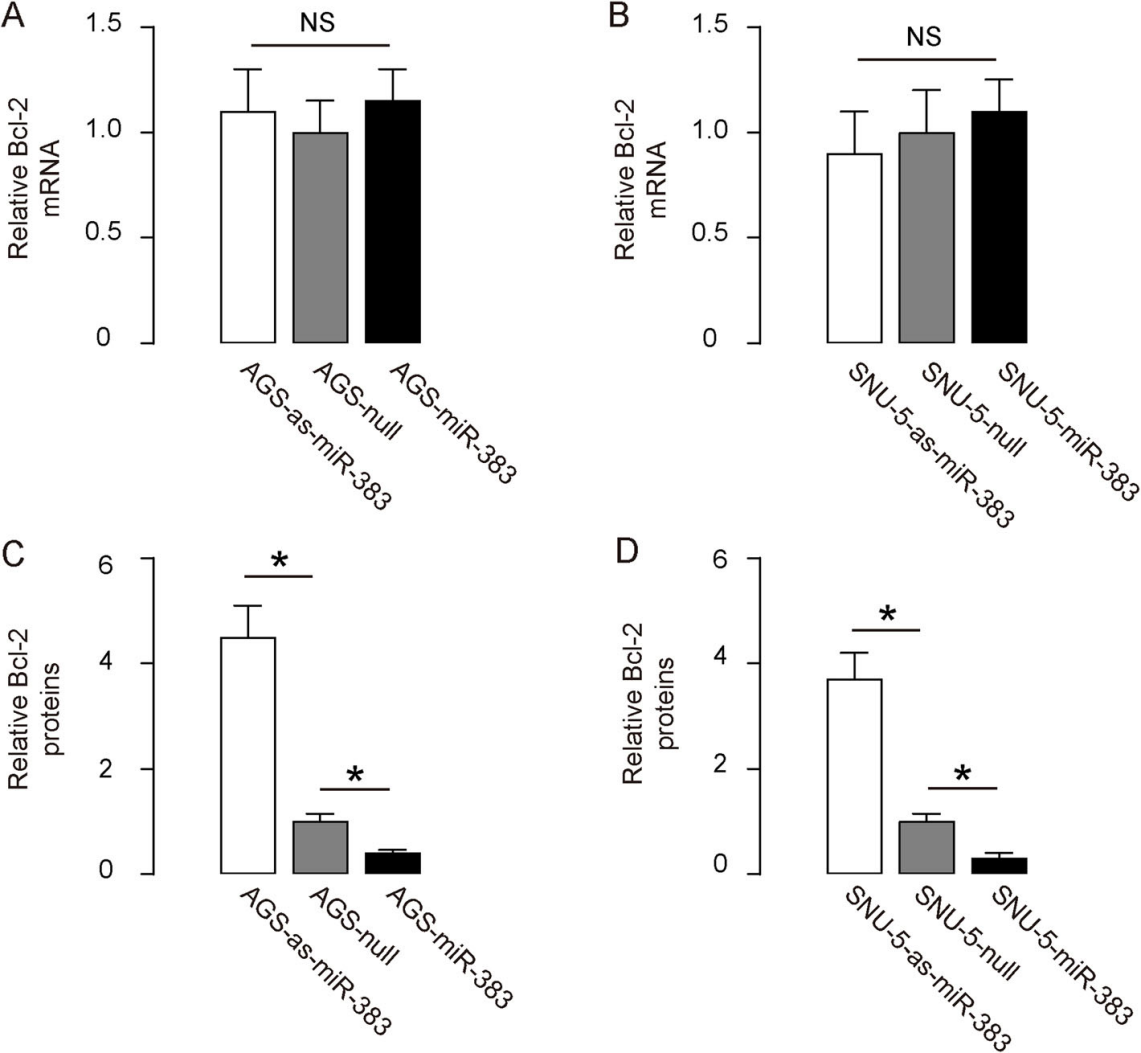
Post-transcriptional control of miR-383 on Bcl-2. **a-b** RT-qPCR for assessing Bcl-2 mRNA levels in miR-383-modified AGS cells (**a**) and miR-383-modified SNU-5 cells (**b**). **c-d** ELISA for Bcl-2 protein levels in miR-383-modified AGS cells (**c**) and miR-383-modified SNU-5 cells (**d**). Values were normalized to null-transfected cells (=1). NS: non-significant. *p < 0.05. N = 5 (each experimental group contains 5 repeats)

We found that altered miR-383 in either AGS cells (Fig. 3a) or SNU-5 cells (Fig. 3b) did not change Bcl-2 mRNA levels. However, miR-383 overexpression significantly downregulated Bcl-2 protein, but miR-383 depletion significantly upregulated Bcl-2 protein in both lines, quantified by ELISA (Fig. 3c-d). Hence, miR-383 inhibits Bcl-2 mRNA translation in cells of GC, without leading to mRNA degradation.

### MiR-383 increases 5-FU-induced apoptosis of GC cells

**Fig. 4:**
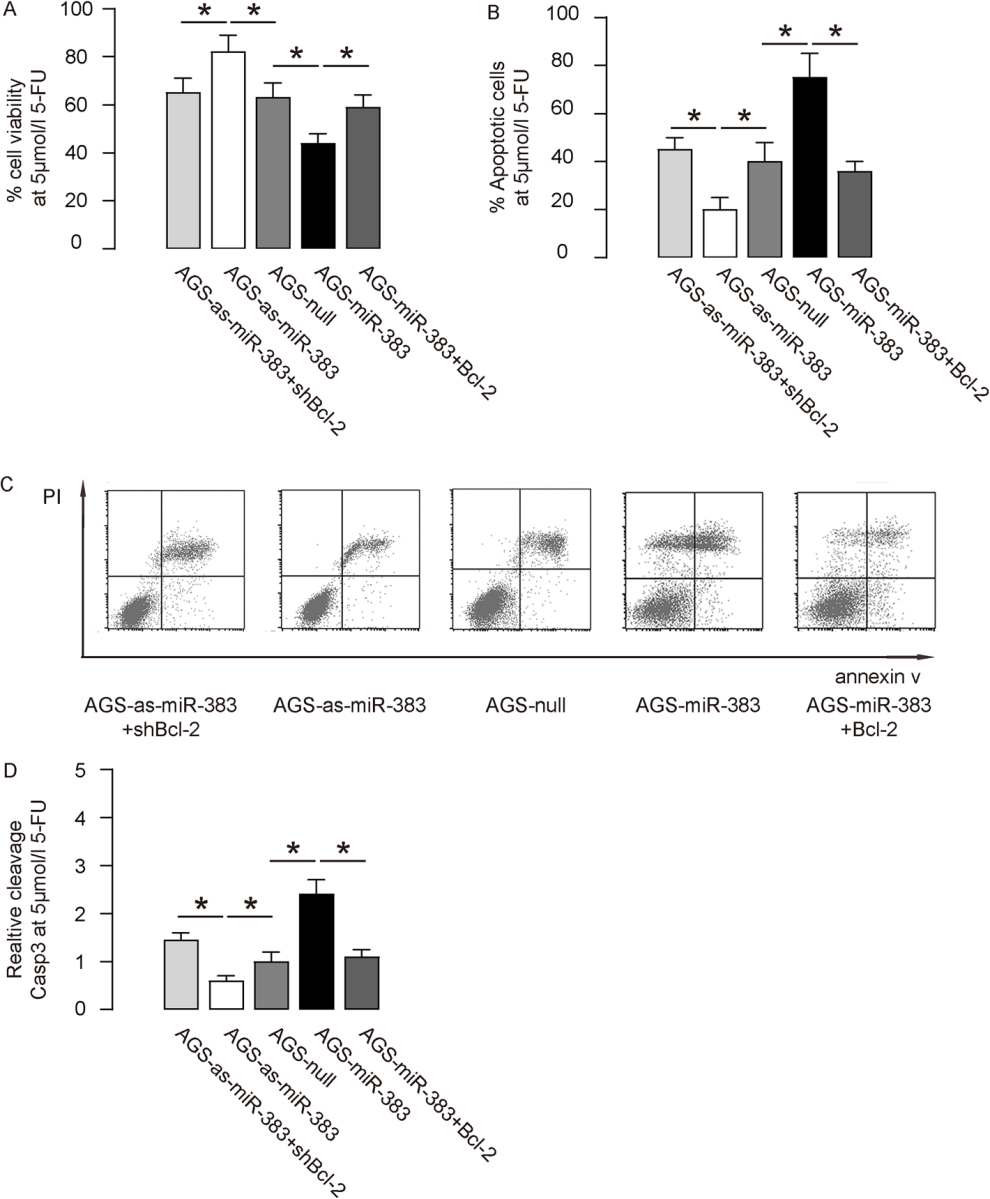
MiR-383 enhances AGS cell apoptosis induced by 5-FU. **a** An CCK-8 assay to determine the effects of miR-383 on AGS cell viability at presence of 5 μmol/l 5-FU. (B-C) Annexin V assay on miR-383-altered AGS cells, shown by quantification (**b**), and by representative flow charts (**c**). (**d**) ELISA for cleavage Casp3. Values were normalized to null-transfected cells (=1). NS: non-significant. *p < 0.05. N = 5 (each experimental group contains 5 repeats)

**Fig. 5:**
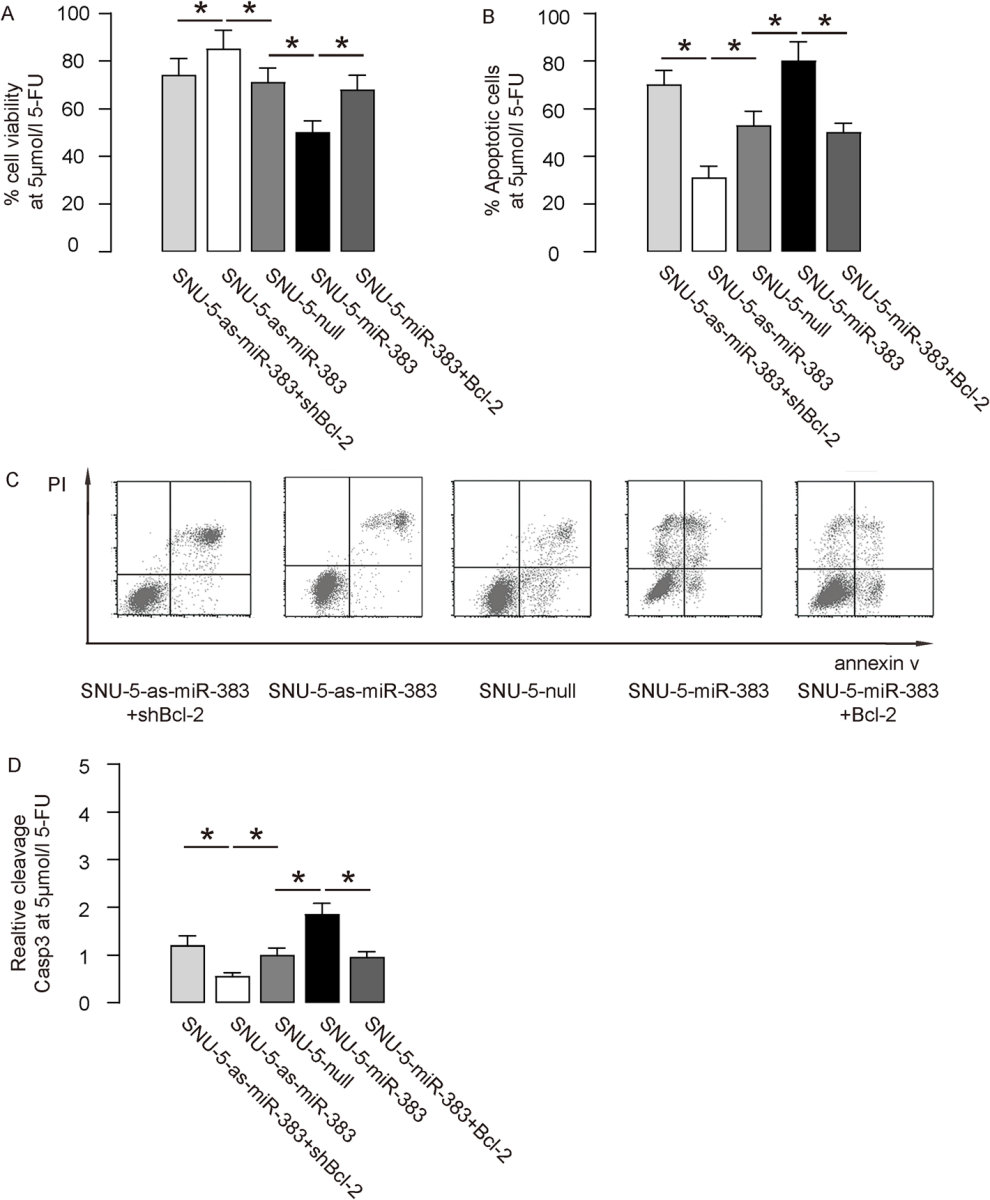
MiR-383 enhances SNU-5 cell apoptosis induced by 5-FU. **a** An CCK-8 assay to determine the effects of miR-383 on SNU-5 cell viability at presence of 5 μmol/l 5-FU. **b-c** Annexin V assay on miR-383-altered SNU-5 cells, shown by quantification (**b**), and by representative flow charts (**c**). **d** ELISA for cleavage Casp3. Values were normalized to null-transfected cells (=1). NS: non-significant. *p < 0.05. N = 5 (each experimental group contains 5 repeats)

To evaluate whether miR-383 may play a role in chemoresistance of GC, first we investigated the effects of altered miR-383 on cell viability of 5-FU challenged GC cells in an assay of CCK-8. We found that miR-383 overexpression decreased cell viability of AGS cells, which was abolished by overexpression of Bcl-2 (Fig. 4a). Moreover, miR-383 depletion increased cell viability of AGS cells, which was abolished by depletion of Bcl-2 (Fig. 4a). In FACS-apoptotic assay, we found that miR-383 overexpression increased cell apoptosis in AGS (Fig. 4b-c) cells, which could be abolished by overexpression of Bcl-2 (Fig. 4b-c). On the other hand, miR-383 depletion decreased cell apoptosis in AGS (Fig. 4b-c) cells, which could be abolished by depletion of Bcl-2 (Fig. 4b-c). The levels of cleavage Casp3 were as well determined in these conditions, showing that the altered apoptosis likely correlated with the levels of cleavage Casp3 (Fig. 4d). Identical experiments were applied to SNU-5 cells, showing nearly same results (Fig. 5a-d). Thus, miR-383 increases 5-FU-induced apoptosis of GC cells through Bcl-2.

### Similar results are obtained from published database

**Fig. 6:**
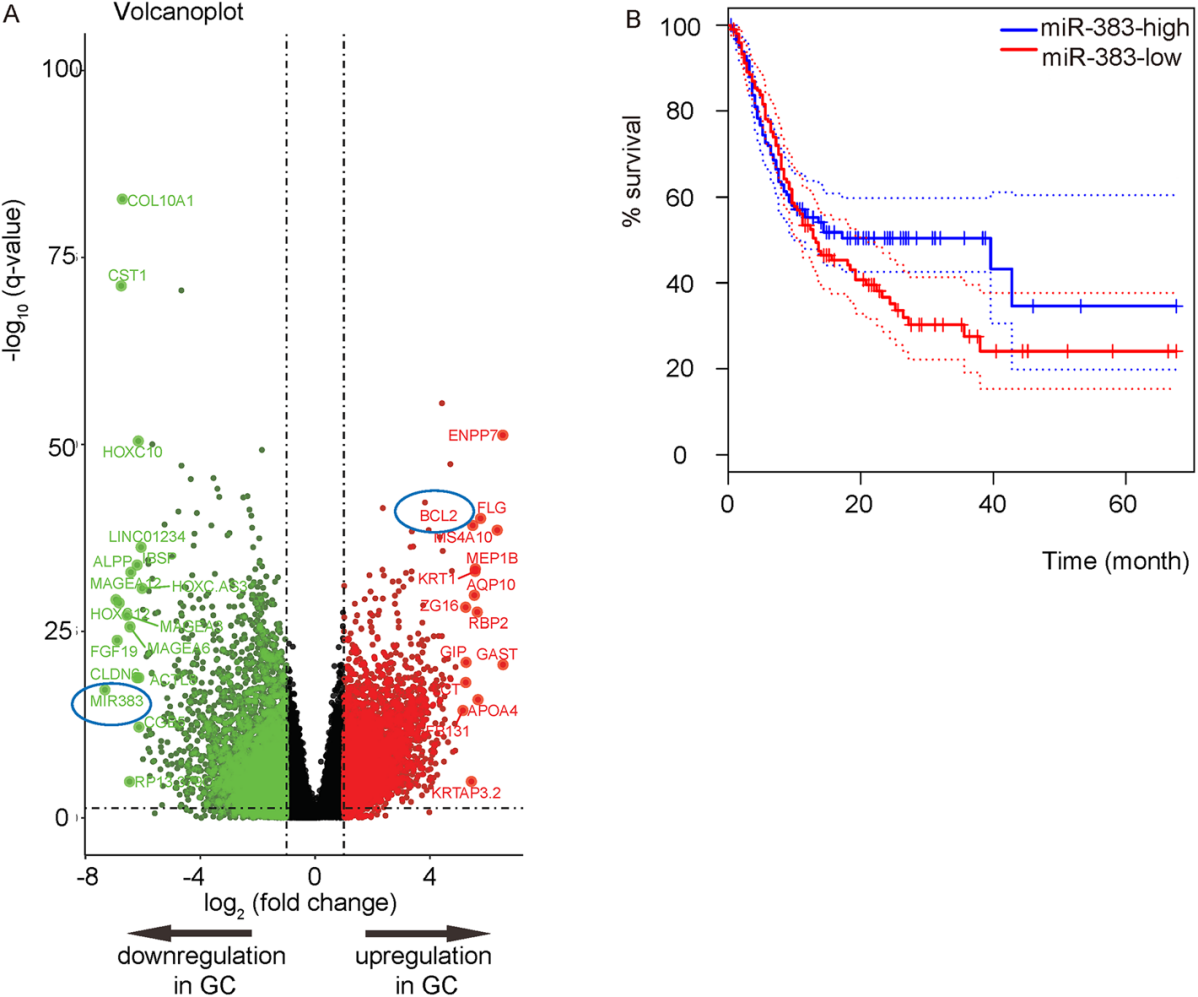
Bioinformatics analyses confirm the regulatory axis of miR-383/Bcl-2 in GC. **a-b** Volcano image (**a**) and survival curve for miR-383 (**b**) for GEO database GSE107754, GPL4133, GPL570 and GPL5639 to compare gene profiling of GC cells to NT cells. Data were analyzed by R language. *p < 0.05. *N* = 463

Finally, we performed GEO database mining and bioinformatics analyses to validate our finding. We reanalyzed the data from some selected public database GC specimens. The Limma R package was used to identify genes that were significantly altered in GC, compared to NT. Shown in a Volcano map (Fig. 6a), expression of miR-383 was significantly low, while expression of Bcl-2 was significantly high in GC (Fig. 6a). Moreover, a similar overall survival curve was detected based on differential miR-383 levels (Fig. 6b).

## Discussion

MiRNAs play critical role in different stages of GC [19–22]. Therefore, understanding of the role of the aberrant expression of miRNAs in the carcinogenesis of GC might could contribute to decipher the mechanisms underlying progression of GC.

The resistance of cancer cells to chemotherapy primarily results from deactivation of apoptotic proteins and the aberrant activation of anti-apoptotic factors in cancer cells. We examined earlier research and found that Bcl-2 activation is possibly a key reaction factor in cells of GC in 5-FU treatment, including activation of apoptosis-association proteins. Indeed, here we found that the levels of cleavage Casp3 seemed to alter secondary to miR-383-regulated Bcl-2, consistent with previous reports showing that Bcl-2 is a direct regulator for Casp3 cleavage to drive the progression of apoptosis [23]. Bcl-2 inhibits these pro-apoptotic proteins efficiently, but the Bcl-2 activation during chemotherapy in different tumor cells applies different mechanisms.

Here, we found some candidate miRNAs that target Bcl-2 by sequence matching in bioinformatics analyses, and we detected a major decrease specifically in miR-383 in specimens of GC among other miRNAs, comparing with the paired non-tumor tissue. Interestingly, miR-383 has been shown to inhibit retinal pigment epithelial cell viability and promote apoptosis and ROS formation likely through Bcl-2 and peroxiredoxin 3 [14]. In another study, miR-383 upregulation was shown to protect hippocampal neuron from undergoing apoptosis in response to propofol via Bcl-2 [15]. Very recently, cell-cycle-related and expression-elevated protein in tumor was found to transcriptionally enhance cyclin D1 expression to promote colorectal carcinoma cell replication likely by miR-383 [24]. Moreover, miR-383 was shown to inhibit GC growth by targeting cancerous inhibitor of PP2A [25], or by targeting HDAC9 expression [26]. In our study, we detected a novel target, Bcl-2, for miR-383 in GC cells. Most critically, our data showed a strong relevance of miR-383/Bcl-2 with prognosis of GC.

## Conclusion

MiR-383 inhibition enhances GC cell survival through Bcl-2 activation during chemotherapy. Mechanistically, the downregulation of miR-383 likely results in increased translation of Bcl-2 protein, which mediates anti-apoptotic cell death against the effects of 5-FU. It may be promising to check whether re-expression of miR-383 in GC cells could substantialize chemotherapy-induced apoptosis.

## Supplementary Information

**Additional file 1.**

## Data Availability

All data are included in the published manuscript and online supplementary material. The analysis on the public database used GEO database GSE107754, GPL4133, GPL570 and GPL5639.

## Abbreviations

3′-UTR: 3′-untranslated region
5-FU: Fluorouracil
Bcl-2: B-cell lymphoma 2
Casp3: Caspase 3
FBS: Fetal bovine serum
GC: Gastric carcinoma
HDAC9: Histone Deacetylase 9
miR-383: microRNA-383
NT: Non-tumor gastric tissue
PBS: phosphate-buffered saline
PP2A: Protein Phosphatase 2A
RT-qPCR: Quantitative RT-PCR

## References

[1] Murai K, Takizawa K, Shimoda T, Fujii S, Sugino T, Yoshida M, Kawata N, Tanaka M, Kakushima N, Terashima M, et al. Effect of double-layer structure in intramucosal gastric signet-ring cell carcinoma on lymph node metastasis: a retrospective, single-center study. Gastric Cancer. 2019;22(4):751–8. 10.1007/s10120-018-00905-9 30523555

[2] Lin Y, Hu D, Zhou Q, Lin X, Lin J, Peng F (2018). The fasting blood glucose and long non-coding RNA SNHG8 predict poor prognosis in patients with gastric carcinoma after radical gastrectomy. Aging.

[3] Li DH, Pan ZK, Ye F, An HX, Wu JX (2014). S-1-based versus 5-FU-based chemotherapy as first-line treatment in advanced gastric cancer: a meta-analysis of randomized controlled trials. Tumour Biol.

[4] Lu ZM, Luo TH, Nie MM, Fang GE, Ma LY, Xue XC, Wei G, Ke CW, Bi JW (2014). Influence of ERCC1 and ERCC4 polymorphisms on response to prognosis in gastric cancer treated with FOLFOX-based chemotherapy. Tumour Biol.

[5] Wu XJ, Yuan P, Li ZY, Bu ZD, Zhang LH, Wu AW, Zong XL, Li SX, Shan F, Ji X (2013). Cytoreductive surgery and hyperthermic intraperitoneal chemotherapy improves the survival of gastric cancer patients with ovarian metastasis and peritoneal dissemination. Tumour Biol.

[6] Kim SG, Jong HS, Kim TY, Lee JW, Kim NK, Hong SH, Bang YJ (2004). Transforming growth factor-beta 1 induces apoptosis through Fas ligand-independent activation of the Fas death pathway in human gastric SNU-620 carcinoma cells. Mol Biol Cell.

[7] Cui Y, Chen J, He Z, Xiao Y (2013). SUZ12 depletion suppresses the proliferation of gastric cancer cells. Cell Physiol Biochem.

[8] Mei Q, Li F, Quan H, Liu Y, Xu H (2014). Busulfan inhibits growth of human osteosarcoma through miR-200 family microRNAs in vitro and in vivo. Cancer Sci.

[9] Wang F, Xiao W, Sun J, Han D, Zhu Y (2014). MiRNA-181c inhibits EGFR-signaling-dependent MMP9 activation via suppressing Akt phosphorylation in glioblastoma. Tumour Biol.

[10] Liu G, Jiang C, Li D, Wang R, Wang W (2014). MiRNA-34a inhibits EGFR-signaling-dependent MMP7 activation in gastric cancer. Tumour Biol.

[11] Di Leva G (2013). Croce CM: **miRNA profiling of cancer**. Curr Opin Genet Dev.

[12] Pereira DM, Rodrigues PM, Borralho PM, Rodrigues CM (2013). Delivering the promise of miRNA cancer therapeutics. Drug Discov Today.

[13] Zhou X, Xia Y, Su J, Zhang G (2014). Down-regulation of miR-141 induced by helicobacter pylori promotes the invasion of gastric cancer by targeting STAT4. Cell Physiol Biochem.

[14] Jiang Y, Sang Y, Qiu Q (2017). microRNA-383 mediates high glucose-induced oxidative stress and apoptosis in retinal pigment epithelial cells by repressing peroxiredoxin 3. Am J Transl Res.

[15] Wang X, Ding G, Lai W, Liu S, Shuai J (2018). MicroRNA-383 upregulation protects against propofol-induced hippocampal neuron apoptosis and cognitive impairment. Exp Ther Med.

[16] Wei C, Gao JJ (2019). Downregulated miR-383-5p contributes to the proliferation and migration of gastric cancer cells and is associated with poor prognosis. PeerJ.

[17] Azarbarzin S, Feizi MAH, Safaralizadeh R, Kazemzadeh M, Fateh A (2017). The value of MiR-383, an Intronic MiRNA, as a diagnostic and prognostic biomarker in intestinal-type gastric Cancer. Biochem Genet.

[18] Coronnello C, Benos PV (2013). ComiR: Combinatorial microRNA target prediction tool. Nucleic Acids Res.

[19] Alessandrini L, Manchi M, De Re V, Dolcetti R, Canzonieri V. Proposed Molecular and miRNA Classification of Gastric Cancer. Int J Mol Sci. 2018;19(6):1683. 10.1186/s12885-020-07711-6. 10.3390/ijms19061683 PMC6032377 29882766

[20] Hao NB, He YF, Li XQ, Wang K, Wang RL (2017). The role of miRNA and lncRNA in gastric cancer. Oncotarget.

[21] Shin VY, Chu KM (2014). MiRNA as potential biomarkers and therapeutic targets for gastric cancer. World J Gastroenterol.

[22] Hua HB, Yan TT, Sun QM (2014). miRNA polymorphisms and risk of gastric cancer in Asian population. World J Gastroenterol.

[23] Donovan M, Cotter TG (2004). Control of mitochondrial integrity by Bcl-2 family members and caspase-independent cell death. Biochim Biophys Acta.

[24] Li J, Smith AR, Marquez RT, Li J, Li K, Lan L, Wu X, Zhao L, Ren F, Wang Y (2018). MicroRNA-383 acts as a tumor suppressor in colorectal cancer by modulating CREPT/RPRD1B expression. Mol Carcinog.

[25] Li X, Yuan J, Cao Q, Xie A, Chen J (2020). MicroRNA3835p inhibits the proliferation and promotes the apoptosis of gastric cancer cells by targeting cancerous inhibitor of PP2A. Int J Mol Med.

[26] Xu G, Li N, Zhang Y, Zhang J, Xu R, Wu Y (2019). MicroRNA-383-5p inhibits the progression of gastric carcinoma via targeting HDAC9 expression. Braz J Med Biol Res.

