# Supplementary figures and images for: Translational control of Bcl-2 promotes apoptosis of gastric carcinoma cells
Source: BMC Cancer. 2021 Jan 5;21:12. doi: 10.1186/s12885-020-07711-6 (PMC7786514; doi:10.1186/s12885-020-07711-6)

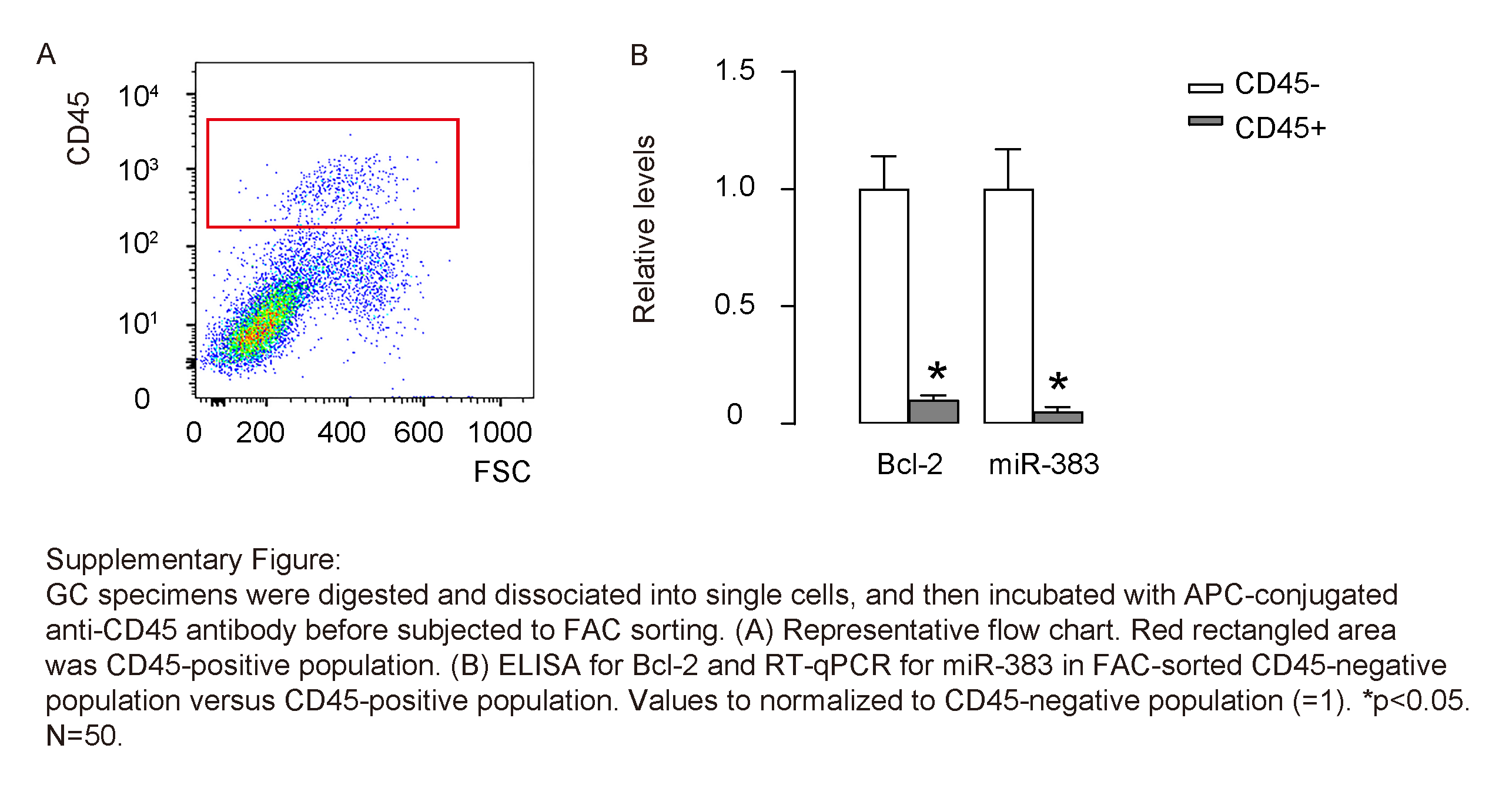

Supplement: Supplementary file 1 — Additional file 1. [file 12885_2020_7711_MOESM1_ESM.tiff]
